# The need to (climate) adapt: perceptions of German sports event planners on the imperative to address climate change

**DOI:** 10.3389/fspor.2024.1505372

**Published:** 2024-12-23

**Authors:** Kim Werner

**Affiliations:** Faculty of Business Management & Social Sciences, Hochschule Osnabrück, University of Applied Sciences, Osnabrück, Germany

**Keywords:** sports events, event planning, climate change, climate adaptation, climate adaptation measures

## Abstract

**Introduction:**

While the impact of anthropogenic climate change on sports and the subsequent need for adaptation to evolving climatic conditions are acknowledged, there remains a notable paucity of scientific inquiry within the realm of sports and sports event studies specifically addressing climate change and its ramifications for event planning and management. Existing studies predominantly stem from health, medical, weather and climate science and mostly focus on mega-events and elite athlete contexts. Moreover, they often only focus on one specific impact (e.g., extreme heat) without providing a comprehensive summary or overview of all eminent impacts, resulting risks and potential adaptation strategies. This study aims to explore how (German) sports events are impacted by climate change and identify measures for organizers to address these impacts.

**Methods:**

Following a comprehensive literature review, semi-structured interviews with event planners and organizers in Germany were conducted, ranging from small local events to weekly league competitions to national championships and major events.

**Results:**

The findings demonstrate that climate change adaptation is not yet a primary focus in the German sports event context. While some planners, especially those of large-scale events, have started implementing adaptation measures, others are only beginning to address the issue.

**Discussion:**

The study discusses the challenges that sports event organizers face in adjusting to the adverse effects of climate change and also examines specific adaptation strategies. The paper emphasizes the imperative for organizers to incorporate climate adaptation measures more effectively into routine event planning and management processes, and provides practical guidelines to achieve this integration.

## 1 Introduction

The advancing anthropogenic climate change has an increasing impact on sports, for example through rising temperatures, bushfires, deteriorating air quality, melting ice and snow, storms or flooding [e.g., (1–4)]. A study from Goldblatt (2) demonstrated that in the coming years, almost all types of sports will be confronted with severe climate change-related impacts. For example, a quarter of the English league football teams will be threatened with flooding each season by 2050 (2). Similarly, it is predicted that only two ski resorts will remain open by 2050 in Germany (5). However, despite a growing body of research on the impacts of climate change across various sports (6–8), there is still a significant lack of scientific studies focusing specifically on sports *events*, i.e., studies that address climate change and its implications for event planning and management (9, 10). Most research stems from health, medical, weather and climate science, rather than from a management, planning and operational perspective [e.g., (4, 8, 11, 12)]. Existing research has also predominantly concentrated on outdoor events, with a particular emphasis on winter sports events (13–15), major and mega-events and elite athlete contexts [e.g., (6, 16–18)]. In contrast, analyses of smaller regional and local sports events have received rather limited attention to date. In addition, existing studies appear to have focused on a selected number of countries, seemingly those in which sports events have already been somewhat affected by climate change in one way or another (for example, through extended heatwaves, forest fires, or the melting of snow and ice), such as the US, Canada, Australia and Austria [e.g., (14, 16, 19–24)]. Finally, the event industry as a whole lacks a unified approach among stakeholders to comprehensively understand and mitigate the adverse effects of climate change from a planning and organizational perspective and to devise strategies for adaptation (9, 10, 25).

This study uses a qualitative approach to critically analyze and discuss the perceptions of German sports event organizers on how climate change affects the planning and organization of sports events in Germany. The study consists of two steps. Firstly, a comprehensive literature review was conducted to analyze the impacts of climate change on sports events in general as well as existing and potential measures for organizers to adapt to these impacts. Secondly, semi-structured interviews with event planners and organizers from sports associations and clubs organizing events of different sizes and forms in Germany—from small local and regional sports events to weekly league competitions (e.g., German Bundesliga) to national championships and major events (e.g., UEFA EURO 2024)—have been conducted. This research is guided by the following research questions:

 (1) How are sports events affected by climate change?
 (2) What are the specific measures by sports event organizers and sports organizations to address these impacts?
 (3) What is the overall status quo of climate adaptation in the German sports events industry?

Overall, this paper aims to spark a discussion on how sports event organizers can adapt their operations to tackle the challenges posed by climate change. Instead of concentrating on a single impact like extreme heat, it provides a comprehensive overview of various climate change-related impacts and potential adaptation strategies. Recognizing that the vast amount of specific scientific information from climate, weather, health, and medical sciences can overwhelm event organizers, especially those of smaller events, this paper seeks to offer practical, hands-on guidelines and recommendations. These are designed to help sports event organizers strategically incorporate climate adaptation measures into their future planning processes.

## 2 Literature review

Climate change significantly impacts sports, thereby affecting athletes, spectators, volunteers and other stakeholders (3, 26). While research in this context is increasing [e.g., (7, 27, 28)], Schneider and Mücke [(8), p. 12] argued that it is “… surprising that the issue of climate change has received comparatively little attention to date in the field of sports science.” Existing research mostly comes from the area of sports medicine and health studies [e.g., (8, 12, 29)].

Schneider and Mücke (8) distinguished between indirect and direct health impacts as a result of climate change. Direct impacts include heatwaves (leading to, for example, heat stress, hyperthermia), ultraviolet (UV) radiation (increasing the risk for skin cancer) and other extreme weather events (e.g., lightning strikes, rockfalls, avalanches, flooding). Indirect impacts refer to air pollutants (e.g., high ozone levels, respiratory stress caused by wildfires), allergens (e.g., extended pollen seasons) as well as increasing incidences of bacteria and viruses. Orr (7) investigated the impacts on sports due to extreme heat, wildfires, bad air quality, flooding, diminishing ice and snow as well as the loss of sports fields and grounds due to increasing erosion challenges as a result of climate change. Climate change also influences the suitability of cities for hosting events (15, 30), affects existing and future sports infrastructure (31) and alters participation patterns in outdoor activities (3, 27). However, research on climate change-related impacts on sports remains limited, and more comprehensive studies are needed to fully understand the implications and develop effective adaptation strategies (3, 27).

The events literature, in particular the literature related to event management and planning, has paid even less attention to the topic, with a scarce amount of studies being published in this area [e.g., (9, 10, 25)]. Mair [(32), p. 7] highlighted that “much of the knowledge required to assist outdoor event organisers to adapt to climate change remains incomplete.” Moreover, the industry itself lacks coordinated efforts by stakeholders to understand the negative impacts and devise managerial and operational solutions for adapting to climate change (9, 25). Werner et al. (10) identified the influencing factors that affect the acceptance of climate adaptation measures among decision-makers in the event industry. They found a high level of awareness of the impacts of climate change on events. However, the interest in the topic among key actors and decision-makers appeared to be low and a wide variety of barriers (e.g., the lack of monetary and human resources) impeded the strategic implementation of specific measures.

The following chapters seek to (a) provide a comprehensive summary of climate change-related impacts in the context of sports events, with a special focus on the managerial and organizational event perspective. The paper then intends to (b) offer an overview of existing and potential measures to better adapt sports events to the consequences of climate change.

### 2.1 Climate change-related impacts on sports events

#### 2.1.1 Extreme heat and rising temperatures

In contemporary research on sports and sports events, thermal stress and extreme heat are the most frequently analyzed impacts (3, 33). Thermal risks can be defined as the probability of suffering from heat exhaustion or heat illness during play or while spectating at sports competitions. They depend on environmental (e.g., heat) and personal factors, such as age, physical fitness, or health conditions (34). The primary health consequences of thermal stress include heat exhaustion, heat stroke, and hyperthermia [e.g., (8, 35–37)]. Due to their prolonged exposure and physical exertion athletes are at higher risk for heat-related illnesses compared to event spectators or the general population (38, 39). Heat strokes are particularly dangerous and may lead to the loss of vital organs or even an athlete's death. Quick medical responses and good contingency plans are therefore needed to minimize the inherited dangers (7). Extreme temperatures also compromise athlete endurance (40) and can lead to heightened fatigue, strain on cardiovascular systems, as well as reduced motivation among athletes (41, 42). In event contexts, however, the needs of supporting staff, as well as those of “ordinary” spectators, also need to be considered. This includes coaches, referees, volunteers, technicians, caterers, and other supporting staff. Of particular concern in this context are vulnerable groups such as children, seniors, individuals with disabilities and people with health impairments (8).

Major and mega sports events in particular have been used to examine heat exposure on athletes and spectators, for example the Tokyo Olympic Games [e.g., (35, 37, 43, 44)], the 2022 FIFA World Cup in Qatar [e.g., (45)] as well as—very recently—the Paris Olympic Games 2024 [e.g., (6, 46)]. Beyond these events, the impacts of thermal stress were investigated in specific sports types such as cricket, cycling, baseball, tennis, American football, canoeing and kayaking, athletics, marathons, triathlons, aquatics, swimming, mountaineering and soccer [e.g., (2, 4, 36, 47–50)].

Previous studies have also highlighted the impacts of rising temperatures on tourism. For example, Falk (51) suggested a positive effect on visitor numbers up to a certain temperature threshold, beyond which a declining trend is observed. As such, moderate temperature increases may render previously unsuitable locations ideal for hosting events due to more favorable and appealing weather conditions (52). For instance, northern Central Europe and Nordic countries could become more attractive for both attendees and event organizers as temperatures rise (53). Contrarily, excessively high temperatures may render certain locations unsuitable for events. For example, Smith et al. (54) projected weather averages in cities across the Northern Hemisphere in the year 2085 in July and August (i.e., the months of the Summer Olympics) to determine how many could still host the Olympic Games. Among 645 cities with a population of 600,000 inhabitants (regarded as necessary to become a host city due to the logistics required), only 33 were still regarded as suitable. As such, rising temperatures and more frequent heatwaves will have an increasing influence on the suitability of future sports event hosts and destinations. Extreme heat also impacts fan engagement by deterring attendance, thus affecting ticket sales and overall engagement with the sport (55). Additionally, high temperatures can degrade playing surfaces, such as turf or track conditions, leading to safety concerns but also to venues, pitches, fields and other grounds becoming unsuitable for use or requiring costly refurbishments (56).

Moreover, climate change is anticipated to exacerbate the frequency and intensity of droughts, thereby affecting health and well-being through compromised water quality, hygiene, sanitation, food security, and air quality (57). The European Commission's Joint Research Centre warned of severe droughts in almost half of the EU and UK territory, highlighting impending water scarcity and competition among users (58, 59). This could result in a rise of large-scale opposition against sports events within communities and among the general population in the future—since these events put additional pressure on already limited water resources. Droughts also affect rivers’ rates of flow which negatively impacts multiple riverine sports events (e.g., kayaking, canoeing, rowing) (2).

#### 2.1.2 Ultraviolet radiation

Outdoor sports participants and spectators are significantly exposed to high levels of UV radiation, which is well-documented to be associated with the development of melanoma and other forms of skin cancer (38, 60, 61). Several factors contribute to high UV exposure in outdoor sports, such as altitude, reflection from snow and ice, and exercise-induced immunosuppression (62, 63). Previous studies have investigated the negative impacts of high UV exposure levels in sports types such as running, triathlon, cycling, skiing, tennis, and hiking [e.g., (62–68)]. However, many athletes still do not use adequate sun protection measures (69). Research indicates that athletes frequently receive UV doses during competition that exceed recommended exposure limits (70). This exposure also impacts eye conditions and may lead to cataracts and pterygium, which can impair athletes’ vision and performance in the long term (63, 71). Episodic sunburn, caused by short-term exposure to intense sunlight, is another risk factor that also often affects spectators and supporting staff.

#### 2.1.3 Extreme weather

Extreme weather incidents, characterized by severe thunderstorms, lightning strikes, heavy rainfall, flooding and strong winds (including hurricanes, tornados, etc.), have increasingly severe implications for outdoor sports events (38). The impact of such weather incidents largely depends on their duration but often results in interruptions or cancellations of events due to safety concerns for athletes and spectators, as well as operational constraints. Also, travel plans for teams, officials and spectators can be disrupted which leads to scheduling and logistical challenges [e.g., (72, 73)]. In addition, media coverage of these cancellations or severe weather-related incidents can negatively affect future event attendance (16, 21, 52). In this context, Giddy (74) analyzed the Cape Town Cycle Tour which was cancelled due to extreme weather, eliciting mixed reactions from participants. While most felt that the weather conditions warranted cancellation, others raised concerns as to how the cancellation was managed. In addition, many felt that the organization of the race needed to be rethought due to numerous negative weather experiences in recent years (74).

Heavy rainfall can cause further natural disasters and damage sporting and event infrastructure as well as operational facilities, leading to significant economic losses for event organizers and local governments (75). It can also cause increased wear and tear on sports facilities, thus necessitating regular inspections and more frequent maintenance, thereby affecting their availability and condition for events (76). Poor field conditions, such as muddy or uneven surfaces, also heighten the risk of injuries (8). Lightning and severe thunderstorms present fatal risks that require strict adherence to safety guidelines as well as awareness and training by medical personnel and event staff (77). Climate change exacerbates these challenges, with more frequent extreme weather incidents threatening the viability of outdoor sports events in the future (78). This also poses significant risks to supply chain stability of events (79). Extreme weather incidents, such as hurricanes and floods, can drastically disrupt transportation networks, leading to shipment delays and higher costs.

Finally, the risk of extreme weather can lead to higher insurance premiums for event organizers, affecting financial planning and budgets (10, 39, 80). Organizers may also face increased liability risks due to injuries or accidents caused by extreme weather, prompting the implementation of more stringent safety measures (81).

#### 2.1.4 Air pollution and air quality

Residents of urban areas are frequently exposed to harmful effects from air pollutants (38, 82), yet, most international sporting events are often held in large cities. Increased levels of air pollutants, such as sulfur dioxide and ozone, can significantly impact athletic performance and health by causing respiratory issues, skin and eye irritations, and general breathing discomfort (38, 83, 84). Ozone pollution, in particular, is detrimental to both performance and health (85). Ozone exposure can induce symptoms such as coughing, chest pain, breathing difficulty, and decreased lung function, all of which negatively affect athletic performance (86, 87). The combination of exercise and ozone leads to more pronounced bronchoconstriction and reduced ventilatory flow as compared to exposure at rest, with athletes being particularly vulnerable due to their higher ventilation rates during exercise (88–90). For example, Sandford, Stellingwerff and Koehle (91) investigated the impact of ozone pollution on athletes preparing for the Tokyo 2020 Olympics and—given that Japan has some of the highest levels of ozone within the Organisation for Economic Co-operation and Development, OECD (with annual peak values of 65–73 ppb)—recommended athletes to adapt to both heat and ozone as part of a simultaneous environmental strategy. Similarly, Bougault et al. (29) identified ozone to be the most probable air pollutant during the Paris 2024 Summer Olympic Games.

In addition, high pollen levels significantly impact sports performance and cognitive function (29). Several studies have reported that the prevalence of asthma is higher in elite athletes than in the general population and that between 2% and 28% of athletes may suffer from asthma depending on different types of sports and methods of diagnosis (92, 93). Li et al. (94) highlighted the need to monitor pollen levels and improve air quality in Beijing for the 2008 Olympic Games.

Next to ozone and pollen, particulate matter also adds concern due to its increased inhalation during physical activity (95–97). The proximity of sports and event venues to high-traffic areas can exacerbate exposure to this particulate matter (98). Indoor sports environments may also face unique air quality challenges (99). Bralewska, Rogula-Kozłowska and Bralewski (100) investigated air pollution in indoor sports facilities and found the presence of specific substances (e.g., propanol, ethanol derivatives) in indoor air which were influenced by specific indoor sources, such as cleaning activities, hall maintenance, and cosmetics. Poor air quality not only affects athletes but also the general public and can deter spectators from attending sports events, as they may experience discomfort or health issues, resulting in lower attendance and engagement (101).

#### 2.1.5 Water pollution and water quality

Water pollution affects aquatic ecosystems, leading to fish mortality, morphological deformities, and diseases (102, 103) and can therefore also severely impact sports events. Pollutants including pesticides, heavy metals, and sewage contribute to various waterborne diseases (104, 105). Research indicates that swimming in contaminated waters during sports events can result in increased rates of infections such as gastrointestinal or ear infections as well as dermatologic conditions and skin diseases among athletes (106–108).

During the 2016 Olympic Games in Rio de Janeiro, swimmers were advised to “keep their mouths shut”, while sailors and windsurfers had to navigate around trash in the contaminated waters of Rio's Guanabara Bay (109, 110). Similarly, the Paris 2024 Olympic Games received widespread criticism due to water quality issues with the river Seine, where two long-distance races and the swimming legs of the triathlon were hosted (29, 111).

#### 2.1.6 Decreasing snow and Ice reliability for winter sports events

The reliability of snow and ice is crucial for the organization of winter sports events. Previous studies have demonstrated that global warming has decreased snow reliability, thereby increasing the need and costs for artificial snowmaking (14, 16, 112). In certain regions of the Northern Hemisphere, the winter skiing season has already been shortened by approximately three weeks over the last fifty years (38, 113). Adequate snow levels are essential not only for planning and staging winter sports events but also for the perceptions of participants, attendees, sponsors, spectators, and the media (16, 52). As such, declining snow reliability and unfavorable conditions may not only lead to decreasing skier demand (114) but also reduced participation rates among spectators and TV audiences, ultimately affecting the overall popularity of winter sports events. As winter sports become less accessible, there may be a shift in popularity towards other sports and events (50, 115, 116).

While advanced snowmaking technologies have reduced dependency on natural snow and ice, the high investment costs pose a significant burden on event organizers and ski field operators (115). It is estimated that 70% of all ski fields in Austria now rely on snow guns, in parts of South Tyrol already 100%. In the 2019/2020 season, cable car operators in Austria invested a total of 754 million Euros, with 150 million Euros specifically allocated to artificial snow (117). The most recent Olympic Winter Games also heavily relied on artificial snow, with Sochi 2014 accounting for approximately 85%, the 2018 Games in Pyeong Chang for 90% and Beijing 2022 for 100% of artificial snow (7, 118). However, even artificial snow does not survive if the temperatures and ground are too warm (i.e., above approx. five degrees Celsius). The increased demand for artificial snow also requires substantial human and monetary resources, high energy consumption, significant water usage, elevated carbon emissions, and consequently leads to considerable negative environmental impacts (7, 119, 120).

The Olympic Winter Games face particular vulnerability, with projections indicating fewer suitable host locations in the future (15, 30, 112, 121). As such, Scott et al. (15) predicted that only one of the previous 21 cities that hosted the Winter Olympics would be able to provide safe snow conditions by the end of the century: Sapporo in Japan. With rising temperatures in the future, many ski resorts will struggle to provide good and safe snow conditions. Major international competitions may therefore face difficulties in finding suitable venues, potentially leading to an increased number of postponements, cancellations or relocations that disrupt international competitions and schedules (122). Warmer temperatures will also result in icy or slushy conditions, increasing the risk of injuries for athletes during sports competitions and putting additional pressure on the organizers (50, 116).

It is, however, not only diminishing snow that is of concern to winter sports events. Ice sports such as ice hockey, ice skating and “sliding sports” (e.g., bobsleigh, skeleton, luge) are also at risk due to shrinking ice conditions and decreasing ice quality. Comparable to the ski events, this may result in shrinking participation numbers, decreasing popularity, increasing costs for adaptation measures, a rising number of postponements and cancellations as well as a higher level of injuries among athletes [e.g., (7, 123–126)].

#### 2.1.7 Infections and diseases

Schneider and Mücke (8) referred to ecosystem changes as a result of climate change, which increase exposure to infectious agents. As such, climate change influences disease transmission by altering the geographic ranges of disease vectors and shortening pathogen incubation periods (127, 128). The number of disease vectors such as ticks, mosquitos and oak processionary moths, is therefore expected to rise. In the future, athletes, but also spectators of sports events, are at more risk of suffering from vector-borne diseases such as dengue fever, West Nile virus, or Lyme disease (40). Similarly, higher levels of allergic reactions are expected due to more pollen strength (for example, from birch, alder, hazel trees and other plants). In addition, wasp and oak procession moth populations are also likely to significantly increase (129).

#### 2.1.8 (Forest) fires

The deterioration of air quality due to forest fires can lead to respiratory issues in athletes and spectators (7, 50, 130). Additionally, forest fires degrade water quality in streams and lakes, potentially affecting water-based sports (131). Disruptions to sports infrastructures and activities caused by forest fires are also a major issue. Fires can destroy stadiums and training facilities, displace athletes, and interrupt sports events and leagues. Forest fires can also lead to the displacement of communities, affecting local sports teams and organizations as members or previous event organizers may be forced to relocate. Fans may be less likely to attend events in areas affected by forest fires, which impacts the attendance of and overall engagement with sports events in the future (7).

#### 2.1.9 Mental health issues

Climate change poses significant risks to the mental health of both athletes and the general population. Rising temperatures and extreme weather incidents have been linked to increased psychological distress, anxiety, depression, and higher suicide rates (7, 132–134). The mental health of elite athletes mirrors that of the general population, with specific vulnerabilities related to sports factors such as injury and burnout (135, 136). Extreme weather incidents, such as hurricanes and extreme droughts, not only cause physical destruction to event venues and sports facilities, but also lead to significant psychological trauma by displacing individuals from their homes, jobs, family members, and communities (137). However, the intricate relationship between climate change and mental health, particularly within the context of sports and sports events, necessitates further exploration (3, 138).

#### 2.1.10 Summary

Overall, the impacts outlined above suggest a gloomy outlook for the future of sports events.

Sports event organizers may face strong economic challenges to counteract the impacts of climate change and introduce adequate adaptation measures to protect the health and well-being of athletes, spectators and all involved. The impacts of climate change will ultimately result in logistical, programming and other operational changes and might disrupt or change event schedules, sporting calendars and seasons. In this context, brands may reconsider sponsorship of events held in areas strongly affected, further impacting funding and support for events. The impacts will also require updates in the infrastructure of event stadiums, facilities, and grounds. Additionally, the heightened risks from climate change and extreme weather may cause more legal challenges if athletes or spectators sustain injuries or illnesses during events. Sports event organizations may therefore face higher insurance costs to cover the risks, once again impacting their viability (81, 139). The next section will outline potential adaptation measures to counteract the increasing impacts in sports event contexts.

### 2.2 Potential adaptation measures in sports event contexts

Before this study looks at the specific adaptation measures pertaining to each single impact, certain measures can be employed by most—if not all—sports event organizers.

#### 2.2.1 General responses and adaptation measures

A measure easy to implement involves, for example, raising awareness among athletes, spectators, and other participants about potential impacts using tools like apps, posters, flyers, display boards, and websites. Additionally, clear and simple countermeasures –such as staying hydrated and applying sunscreen—should be emphasized to highlight the personal responsibility of everyone involved. Similarly, continuous training and education sessions on the impacts of climate change and on adaptive measures should be conducted for event organizers, staff, and volunteers. This training should be specific to the type of event and its location and might include close cooperation with municipalities, councils, and other authorities to also educate staff there and to learn from each other (7, 8, 28, 129).

In this context, municipalities, councils, local governments and authorities play a significant role since investing in sports and event infrastructure is critical for future-proofing venues (31). This includes, for example, installing water dispensers or tanks, shaded areas or greening the roofs and façades. Event organizers should also invest in materials suitable for extreme weather conditions, such as durable tents and other weatherproof equipment. As outlined earlier, extensive weather forecasting and monitoring is also a measure that can easily be applied (129, 140–142).

Collaboration is another important adaptation measure in the context of sports events and may include collaborating with meteorologists, weather and climate experts, medical doctors and institutions (including dermatologists, psychologists, and other sports medicine specialists), councils, authorities, transport and regulatory offices, urban planners, and architects but also emergency services (including police, fire services, disaster response teams and many more). In addition, partnerships between and among clubs, sports venues, sports federations and associations or sports event organizers can provide support and alternative locations if needed. Such partnerships can also offer access to guidelines, best-practice examples, (legal) advice, and monetary support, thus ensuring informed decision-making (7, 28).

Given the increased risk for disruption and the resulting legal challenges, having sufficient insurance coverage and a good relationship with the insurance company is also vital (81). In addition, clear policies should be established by event organizers regarding when to interrupt, postpone, or cancel events. This includes determining an alternative date for interruptions or cancellations which can be communicated quickly. There should also be a detailed emergency and evacuation plan with clearly defined responsibilities. A robust communication strategy is also essential, which includes information on how to deal with the media, sponsors and other relevant stakeholders (143).

#### 2.2.2 Extreme heat

To minimize the impacts of severe heat, free or additional water, movable water dispensers, and more hydration stations can be provided by event organizers. Other measures include additional cooling areas or tents, access to fridges or cold storage rooms as well as extra retreat areas, tents, or rooms. Cooling aids, such as cold water or ice packs, ice vests, cool towels, hats, headbands, socks, inflatable ice tubes, baths, and misting systems for spectators or athletes can be offered before, during, and after events. Providing free sunhats, umbrellas, parasols, awnings, fans, and sunscreen can enhance comfort and safety. Catering options should include water-rich fruits and vegetables, like melons and cucumbers, along with snacks and drinks that replenish carbohydrates and minerals [e.g., (6, 43, 144–147)].

From an organizational point of view, start times can be postponed to early mornings or evenings, or locations may be changed entirely to more suitable venues. Organizers can also introduce more drinking and cooling breaks (148). In addition, the duration of competitions or race distances could be reduced to decrease athletic loads as well as spending times in extreme heat. In team sports, changing players and referees more frequently could also be an option. Adjustments in scoring or performance metrics may be necessary to account for the challenging conditions (129, 149). However, this requires close collaboration with the respective sports federations or associations.

A “heat orientation plan” as proposed by Schneider (150) including “emergency give-away bags” for spectators, visitors, and participants can be prepared, which also adds new sponsorship opportunities. This bag could contain a water bottle, sunscreen, lip balm, hat, sunglasses, insect repellent, and sanitizers. Medical services, support and care for athletes and first aid stations for spectators are crucial (8, 149). Several sports organizations have already implemented heat policies, such as those enacted by the Union Cycliste Internationale (UCI), the Australian Open Tennis Tournament or the German Football Association (22, 48, 151). A selection of exemplary heat policies and recommendations introduced by different sports associations can be found in Table 1.

**Table 1:** Exemplary heat policies and recommendations introduced by selected sports associations.

Event	Thresholds and measurement	Adaptation measures
Union Cycliste Internationale (UCI) race regulations https://www.uci.org/high-temperature-protocol/2pNk2Cf4VOBGuHBd68jAnK	The risk assessment (basis: Wet Bulb Globe Temperature (WBGT) is expressed in the form of a color code	
	• White zone (WBGT below 15°C), very low risk;	None
	• Green zone (WBGT between 15°C and 17.9°C), low risk;	Warm-up in the shade with fans, skin protection with non-greasy sun creams, choice of light-colored clothing, normal hydration plan.
	• Yellow zone (WBGT between 18°C and 22.9°C), moderate low risk;	Warm-up with ice vests, use of fresh towels, application of strict, individualized hydration plans, distribution of “ice-socks”, supply of ice to the teams during the race.
	• Orange zone (WBGT between 23°C and 27.9°C), moderate high risk;	Adaptation of the start area to keep riders in the shade before the start, protect officials, organizing staff and volunteers from the sun, increase the number of neutral motorbikes providing riders with drinks and ice packs, adapt the rules limiting hydration and cooling in competition.
	• Red zone (WBGT above 28°C), high risk.	Modification of start and finish times, possible neutralization of a section of the race or stage, cancellation of the stage/race
German Football Association (DFB) https://www.dfb.de/news/detail/bei-hitze-gilt-trinken-trinken-trinken-126309/	The DFB Sports Medicine Commission warns of ‘medical risks for players and spectators in extreme heat and makes the following recommendations:	
	If the temperature is above +35°C (or above +32°C if the humidity is above 80 per cent)	Possible postponement of matches (to the evening hours or to another date)
	No threshold	Possible heat/sun protection for spectators: light-colored caps with sunshades
	No threshold	Water supply, large umbrellas/roofs, humidification with water
	No threshold	Possible heat/sun protection for players: Pre-cooling (various methods, water most effective carrier), cold drinks, cap for goalkeeper, adequate clothing for sweat and temperature dissipation, one to two drinking breaks per half-time by the referees.

In this context, the wet-bulb globe temperature (WBGT) index, which combines temperature and humidity, is often used as an indicator and basis for decisions (22, 37, 43). There is, however, no unified approach in terms of weather policies for sports events. As such, single events (e.g., World Athletics Championships, Australian Open) or single sports associations (e.g., UCI, International Association of Athletics Federations) have introduced different measures and heat-related policies to protect athletes and spectators. Racinais et al. (152) criticized the large variety of single policies which use different indicators, do not consider relevant environmental conditions, are hard to understand or implement in practice or are scaled in confusing formats (e.g., a WBGT of 30.6°C was marked orange by the Tokyo Organizing Committee of the Olympic Games but corresponds to a red flag for the World Triathlon and is classified as black by the International Federation of Modern Pentathlon). These aspects make it particularly hard for (smaller) event organizers who—next to the sports-specific aspects—are involved in the general, event-related logistical planning and organization of the event. In addition, temperature thresholds for athletes and participants are different compared to those for the general population such as spectators or volunteers (150).

As part of a working group by the IOC Medical and Scientific Commission and several international federations (IF), Racinais et al. (152) developed recommendations for event organizers to monitor environmental conditions before and during an event, to introduce specific adaptation measures (e.g., ice, shading and cooling); and to remove regulatory and logistical barriers. They highlighted the necessity to “… communicate with the different stakeholders using their own language, including video and social media for athletes, policies for IFs and organizers, medical workshops for clinicians, etc.” [(152), p. 14].

#### 2.2.3 Ultraviolet radiation

To better adapt to UV radiation-related risks, experts advocate for protective strategies that include scheduling events during periods of lower UV intensity (e.g., mornings or evenings), encouraging athletes and spectators to wear appropriate clothing and accessories (e.g., light clothing, long sleeves, sunglasses, hats, etc.), and to apply water-resistant sunscreens (153, 154). Some sports events have successfully introduced sun-safe protocols, which have shown effectiveness in reducing UV exposure among participants and spectators (155). Next to weather forecasts, other methods used to measure UV exposure include personal dosimeters and smartphone-based technologies (156, 157).

Adaptation measures related to UV radiation generally include similar measures compared to those that seek to counteract extreme heat. Competition or performance times can be reduced to limit exposure for athletes and spectators. Providing (branded) sunhats, umbrellas, parasols, awnings, and sunscreen can enhance protection against the sun for athletes, spectators, and volunteers.

#### 2.2.4 Extreme weather

Although the weather itself cannot be controlled, attempts have been made to adapt to the impacts by adopting new technology (e.g., artificial/synthetic playing surfaces that can better absorb precipitation), moving indoors, and insuring against climate-related losses (158). The measures also include infrastructure improvements to stadiums and other event and sporting facilities such as rainwater storage and derivation (31, 72, 74).

In the context of large-scale sports events, such as the Olympics, professional weather forecasting and prediction systems have been used for quite some time to provide guidance to the organizers [e.g., (141, 142, 159, 160)]. Olya (39) demonstrated how mega sports events such as the Tokyo 2020 Olympic Games can benefit from intensive analysis of long-term weather forecasts, thus facilitating optimal event scheduling. Given that extreme weather incidents can occur at short notice, continuous weather monitoring before and during the event is essential. This can be done using simple weather apps or more advanced options such as personalized weather advice and support by private or public institutions such as the German Weather Service or UBIMET [e.g., (161, 162)]. Similarly, outdoor sports events need to establish clear guidelines to ensure the safety of participants and spectators in severe conditions like lightning and high winds (77). This includes a detailed evacuation plan, the provision of shelters to protect athletes and spectators as well as cooperation with emergency services and local authorities. Effective communication with all participants about weather-related decisions is also important (74). Overall, attempts to better adapt to severe weather conditions have been limited by the costs involved, both absolutely and in relation to the perceived benefits, but also by attitudes and lack of interest. For example, Kay and Vamplew (158) found that sports promoters and authorities in Great Britain do not perceive the British weather as extreme and therefore tend to adopt reactive rather than pro-active policies.

#### 2.2.5 Air quality

There are several measures to adapt to the risk of bad air quality, such as limiting outdoor exercises during high pollution periods, monitoring the air quality, wearing N95 masks, and using antioxidant supplements (86, 163). As such, organizers could distribute masks and sunscreen to protect athletes and spectators. Improved weather and pollen forecasting are also crucial (164). Although some guidelines exist concerning air pollution thresholds during physical activity [see (165)], a clear consensus on specific values is lacking. During periods of high pollution, outdoor exercises may be limited by interrupting the event and postponing it to a different time of day or season when air quality is better [e.g., (7, 87, 88, 95, 163, 166, 167)].

#### 2.2.6 Water quality

Continuous monitoring of water quality is an essential measure in this context (106). Detected pollution may necessitate additional cleanup efforts to ensure event safety, thereby impacting the budgets and resources of sports event organizers. Weather monitoring should also be conducted to assess conditions that might impact water safety (e.g., extended periods of rain). Clear policies should be established by the organizers as to when to close waterways to protect participants (1, 7, 26, 108). Guidance could also come from the World Health Organization and the European Union. Both organizations have established guidelines for recreational water quality assessment, focusing on indicators like E. coli and enterococci levels (168, 169).

#### 2.2.7 Infections and diseases

To avoid the spread of infections and diseases, several measures can be implemented: Providing food that is not easily perishable ensures safety and reduces the risk of foodborne illnesses. In this context, hygienic checklists and disinfection facilities are crucial for maintaining cleanliness and safety standards. Distributing insect repellent or hiring technical solutions to control mosquito populations can help minimize the risk of insect bites. First aid stations and medical services should be available, with a particular focus on addressing wound infections, contact with pests (like ticks and oath procession moths), and insect bites (8, 28, 40). Information material and display boards can be used to keep spectators, visitors, athletes, and other stakeholders informed about safety measures and potential risks. The “emergency give-away bag” (see above) proposed by Schneider (150) could also be a valuable measure here.

#### 2.2.8 Mental health issues

Medical services and doctors should be available, including psychologists to address any mental health concerns. Preparing additional retreat areas or tents can provide safe spaces for individuals to rest and recuperate if needed. In addition, clear policies should be established regarding what will happen to the competition and scoring in case of any interruptions, postponements, or cancellations. This ensures transparency and fairness for all participants and reduces pressure on high-performing athletes (7, 133, 135, 136, 170).

#### 2.2.9 Snow/ice

As outlined earlier, advanced snowmaking technologies and scientific projections of future temperature developments have reduced dependency on natural snow (16, 115). It is estimated that 95% of ski resorts now rely on snow guns for at least some of their snow (7). Other measures include snow farming which involves producing a lot of snow during the cold season and storing it under tarps throughout summer for use during the ski season (171). Snow transport—albeit not regarded as a sustainable option—has also been used, for example during the Vancouver 2011 Winter Olympics, to transport snow from higher altitudes via helicopter or truck (7). To ensure the health, safety and well-being of athletes and spectators in times of deteriorating snow and ice quality, a comprehensive risk management approach should be adopted (172). This also involves continuously monitoring not only the weather but also the event locations (e.g., ski fields, ice channels, etc.) and their surroundings to identify potential hazards such as icy conditions, lack of snow or ice, increased levels of mountain, rock or mud slides, and avalanche risks (126, 140, 141, 173). Adjustments to the event can also include changing the start times or reducing the competition time to adapt to weather and environmental conditions. Relocating the event to other areas in higher altitudes or with different climatic conditions are also options (171, 174, 175). However, previous research has pointed to the counterproductive effects of climate adaptation measures on ski tourism (176). For many guests in skiing destinations, the negative effects of adaptation measures outweigh the benefits in two ways: they contribute to the deterioration of nature and landscape aesthetics, and they induce higher prices without real added value. This might be the same for events and could result in a decreasing popularity and attendance of winter sports events in the future. The growing impacts of climate change might eventually render certain destinations unsuitable for hosting winter sports events. Consequently, destinations have already started to substitute traditional winter sports events with alternative tourism activities (e.g., hiking instead of skiing events), or with different types of events (e.g., music events or other sports events) (175, 177).

#### 2.2.10 (Forest) fires

Adaptation strategies in the context of fires particularly include intensive weather and air quality monitoring and close collaboration with emergency services, councils and other government agencies (130). Information materials and display boards can also help to keep spectators, visitors, athletes, and other stakeholders informed about safety measures, evacuation routes and alarms. Preparing an evacuation and communication plan is vital to ensure a swift and organized response in case of an emergency (7, 80, 130).

#### 2.2.11 Choice of venues and locations

An additional adaptation measure in sports event contexts refers to the strategic choice of sports venues and locations. In this context, sports event organizers—who are able to choose their venue or location—should give preference to microclimate-favorable venues and buildings with modern insulation, energy-efficient refurbishments, temperature management options, proper ventilation and air conditioning systems. Additionally, venues and locations need to provide both natural and artificial shading. This can be done through the use of permanently installed structures or movable parasols and awnings but also natural shading (i.e., trees and plants). Furthermore, the locations or venues should offer essential amenities such as water dispensers and sufficient shower facilities. There should be ample cooling areas as well as access to fridges, refrigerators, or cold storage rooms to cool down food and drinks and to provide cooling aids such as ice packs. Lastly, for outdoor events, the proximity of stable buildings or shelters is crucial, as they provide a safe space during prolonged heat but also extreme weather-related incidents such as storms, hail or lightning (8, 28, 129, 150).

In summary, a wide variety of adaptation measures are applicable for sports event organizers and several of them help to minimize the risks for more than one impact. The measures outlined above do not claim to be complete but offer some important (starting) points for event organizers to act and—therefore—mitigate risks. It also needs to be noted that the ultimate scenarios to address these impacts might be postponements, relocations or entire cancellations. As such, it might be necessary to postpone an event entirely to a different season if conditions become too hazardous. Alternatively, moving the event indoors (if possible) or to another location can provide a controlled environment that mitigates the risks. In this context, Mair (25) referred to spatial, temporal and activity (i.e., offering a different event or activity) substitution options. In the worst-case scenario, the event might need to be cancelled altogether (7, 42, 141). However, postponements, relocations and cancellations come at a high financial cost for organizers (including the potential loss of broadcasting and sponsorship revenue), lead to logistical and operational challenges and disrupt international sporting and competition schedules (7).

Following this comprehensive review of impacts and potential adaptation measures, the next chapter describes how climate change affects German sports events, thus addressing research question #3.

### 2.3 Climate change affecting Germany and German sports events

In Germany, the average annual temperature statistically rose by 1.6 degrees Celsius between 1881 and 2022. For the period since 1881, the five hottest years in Germany were recorded from 2000 onwards. This means that temperatures in Germany have risen above the global average (around 1 degree Celsius). This is because land areas generally warm up faster than marine areas. On average, the number of hot days with an average temperature of over 30 degrees per day in Germany has tripled since the 1950s from around 3 days to an average of 9 days per year today. While significant heat waves have increased in frequency and intensity during this time, the average number of freezing days (with a maximum daily temperature below 0 degrees) has fallen from 28 to 19 days per year in this period. For the future, the climate projections for climate scenario RCP8.5^1^ predict an increase in the near-surface air temperature of 3.1–4.7 degrees Celsius in the period 2071–2100 compared to the period 1971–2000 (178). As a response, the German government adopted the “German Strategy for Adaptation to Climate Change” back in 2008 (179). On July 1, 2024, the Climate Adaptation Act was enacted, mandating the development of climate adaptation concepts at all federal levels. The legislation aims to implement comprehensive climate precautions across Germany by utilizing targeted systematic impact analysis and action planning (180).

Climate change also affects sports and sports events in Germany [e.g., (28, 56, 148, 149)]. Schneider (129) proposed an adaptation model called the “sports, clubs and climate change model (SC^3^-pyramid-model)”, which contains a variety of technical-structural (e.g., the greening of roofs and façades), organizational (e.g., additional drinking breaks) and person-related measures (e.g., sports fitness examinations of athletes, medical observations) plus some cross-sectional measures (e.g., training and further education, collaboration). In the context of major sports events, Schneider (150) suggested a “heat orientation plan” for major sports events which contains detailed and valuable recommendations for the organizers of sports events affected by extreme heat (e.g., providing shaded areas, sunscreen, ice, water dispensers, etc.). However, the topic appears to not yet be widely discussed in practice, i.e., between and among sports associations, clubs, athletes, and sports events organizers in Germany. For example, Schneider et al. (33) investigated to which extent the top 66 German sports associations organized in the German Olympic Sports Federation (DOSB) inform amateur and professional athletes about the health effects of climate change on their central websites. They found that only very few of the top associations address sports-specific health risks of climate change on their websites. Only one in six top associations provide information on heatwaves, one in five on UV exposure and one in ten associations discusses extreme weather incidents or infection risks. Risks from ozone and allergens are addressed even less frequently. Given the increasing importance of climate change for athletes and sports organizations, the authors highlighted the need and relevance to become more active. While some associations have already adopted certain recommendations or even policies, the majority have not and there appears to be a lack of a strategic and unified approach among sports associations and sports event organizers in Germany. To further investigate the current overall status quo of climate adaptation in the German sports events industry (see research question #3), semi-structured interviews were conducted. This process is described in the next chapter.

## 3 Methods

This research adopts a constructivist paradigm informed by qualitative methodologies, reflecting a perspective of a socially constructed, complex, and dynamically evolving reality (181). The objective of the study was to generate meaningful insights into the individual experiences of sports events organizers and employees from sports associations in Germany on the impact of climate change on sports events as well as potential adaptation strategies. A total of 13 semi-structured interviews were conducted. The sample included employees from both large German sports organizations and events (such as the Special Olympics Berlin or the German Football Association) as well as small scale regional event organizers (e.g., a local mountain biking event, a triathlon event). A purposive sampling approach was utilized, and all participants were recruited through professional contacts and snowball sampling methods. An overview of all participants is presented in Table 2.

**Table 2:** Overview of interview participants.

No.	Organization/event	Interview partner(s)	Dates	Background information
1	Special Olympics World Games Berlin 2023 (also involved in the Special Olympics National Winter Games, Oberhof/Thuringia 2024)	Chief Operation Officer	17–25 June 2023 (Winter games: 29 January–2 February 2024)	International sporting event for participants with intellectual disabilities from all over the world, organized by the IOC-recognized Special Olympics organization; approx. 7,000 athletes in 26 different sports; closing ceremony in front of the Brandenburg Gate in Berlin with 21.000 participants (Winter Games: 900 athletes in 10 different types of sport)
2	UEFA Euro 2024, host city [name of city confidential]	Member of the host city organizing committee [position confidential for privacy reasons]	14 June–14 July 2024	international football tournament with 24 teams from Europe competing in 9 cities across Germany; the selected host city hosted several matches; next to the stadium, fanzones within the city center offered public screening
3	[Event name confidential] 24 h-mountain bike race	The two principal organizers	Three days (weekend) every year in May (since 2010)	Mountain bike event with adjacent campground in Northwestern Germany; riders can take part individually or in groups; the winner is the rider or team with the most complete laps after 24 h. 780 riders took part in 2024.
4	German Olympic sports confederation, DOSB	Sports facilities and environment officer	Various	The non-governmental umbrella organization of German sport; represents 89,000 sports clubs and 27,000,000 members throughout Germany (including the 16 state sports associations)
5	[Event name confidential] international horse-riding competition	Event manager	Five days; every year at the end of April/beginning of May	Several competitions, including Grand Prix Spécial, Grand Prix Freestyle (both dressage), and a Grand Prix for show jumpers (part of the Riders Tour). In 2024: over 550 horses, 250 participants from 27 nations, 35,000 visitors
6	German Football Association, DFB	Sustainability department	The Bundesliga season runs from the end of August to the beginning of May each year	The governing body of football, futsal, and beach soccer in Germany. A founding member of both FIFA and UEFA, the DFB has jurisdiction for the German football league system and is in charge of the men's and women's national teams.
7	[Event name confidential] triathlon	One of five main organizers	Every year in July/August (since 2010)	Local triathlon event in the Northwestern part of Germany; approx. 160 athletes, and around 80 staff (mostly voluntarily)
8	SV Werder Bremen (German Bundesliga club)	Sustainability coordinator	Bundesliga season: end of August to beginning of May each year	One of the founder members of the German Football Association (DFB); has been German champions four times; 42, 000 members; Stadium: Weserstadium, current capacity 42,100
9	Landessportbund Bremen (State Sports Association of Bremen)	CEO (1) and Coordinator for recreational and competitive sports (2)	Various	The umbrella organization of 430 sports clubs and 50 sports associations in the German Federal State of Bremen with around 155,000 members (representing over 50 different types of sports)
10	[company name confidential] Event technology company	Head of HR/event project manager	Throughout the year	The company is responsible for all technical aspects of a large variety of outdoor sports events, operating in all areas of Germany (e.g., horse riding, running)
11	[company name confidential] Event insurance company	PR manager	Throughout the year	The insurance company offers tailored coverage options for sports associations/federations and their events, including public liability insurance, event cancellation insurance, indemnity insurance, weather index insurance.
12	German canoe association	Head of sustainability & environment	Various	The German Canoe Association is the largest canoeing association in the world. It has around 129,350 members who are organised in around 1,300 clubs and regional associations. Its activities range from alpine white water canoeing, leisurely small river hiking and saltwater tours to sports competitions and events.
13	[Company name confidential] triathlon	One of the three main organizers	Every year in July (since the 1980s)	Local running event in the Western part of Germany; up to 750 participants, different races/distances; participants include professional athletes but also the general public.

The interviews were conducted via Zoom and Microsoft Teams, audio-recorded, and transcribed verbatim. Supplementary notes were taken to capture significant thoughts and observations (182). Key questions included: “How would you say that your event is affected by climate change?”, “Can you provide some specific examples?”, “Are there specific measures that you have implemented already to reduce the negative impacts?” and “What additional measures are you considering for the future?” The interviews were conducted in German and lasted between 31 and 52 min. The author of this paper is bilingual and carried out a three-step process of translating, editing, and proofreading which is “reliable for creating a translation that is true to the original document” [(183), p. 67]. In addition, the transcriptions were sent to the interviewees for verification, to make sure the transcription was a true and accurate reflection of what had been said. This process also minimized the likelihood of researcher-imposed bias (182, 184). Thematic analysis was employed to process and analyze the data obtained from the 13 interviews (185–187). The data analysis process was facilitated by MAXQDA software, which proved to be a useful tool for systematically coding and theming the data (188). An open-coding process was utilized, adhering to Braun and Clarke's (185) step-by-step guide for thematic analysis. In an iterative process, codes and themes were continually revisited and verified (182). The continuous interaction with the data, a thorough process of thematic analysis, and the use of MAXQDA software added salience and depth to the process, prevented premature closure and enhanced trustworthiness (189). With the help of the software, a total of five themes were identified across the dataset (see Table 3). These themes are elaborated upon in the subsequent sections.

**Table 3:** Themes derived from data analysis.

No.	Theme
1	Awareness of climate change-related impacts
2	Specific impacts of climate change
3	Measures implemented to adapt
4	Challenges involved for event organizers
5	Outlook and the future of climate adaptation in sports events contexts

## 4 Findings

### 4.1 Awareness of climate change-related impacts

All interviewees were aware of increasing climate change-related impacts on their sports events, and most had already experienced some of these impacts. Asked about the biggest challenge when organizing his international sports event, one interviewee emphasized:

For us, or all outdoor events literally, the biggest challenge is usually the weather, because you can’t influence it and you’re dependent on it. That’s what an outdoor event lives on—if the weather is good, the event is good.

The interviewees pointed to an increasing frequency of extreme weather incidents that they needed to deal with over the last few years and that they expected to become more common in the future. For example, one participant emphasized “storms …, they're increasing”, while the organizers of an international mountain bike race highlighted:

This year we had … more rain and more extreme rain than the weather models predicted, so like just a bit of rain, a few millimeters, but not what eventually came down and that’s when you have to interrupt a race.

As such, a lively discussion on how to better prepare for the impacts of climate change on sports events was part of every single one of the 13 interviews. It was also emphasized that many local authorities and event organizers are not yet sufficiently prepared for these issues, particularly those organizing smaller events. However, while all interviewees were aware of the impacts of climate change, most interviewees highlighted that the topic has only recently received more attention. For example, the interviewee from the German Olympic Sports Federation (with over 85,000 sports club members, including the 16 German state sports associations) noted:

… we have noticed that the topic of climate adaptation is becoming increasingly important. So sport is affected by climate change and we need to ask ourselves in which areas we need to adapt. We have mainly focused on climate protection so far, so we are still in the early stages when it comes to climate adaptation. But for the last year and a half or so, the topic has increasingly been on our agenda.

In this context, some interviewees also highlighted a certain reluctance to address the topic publicly with other partners or stakeholders, given that some still do not recognize the urgency or necessity of action, especially those who continue to deny climate change. One interviewee mentioned, for example: “… often when you … talk about the topic, you just get some stupid comments like “Yes, but it has always rained before” or “in the past it used to be hot too, that's called summer.””

### 4.2 Specific impacts of climate change

The most frequently mentioned impacts included extreme heat, torrential rain (and resulting flooding) and storms (including thunderstorms/lightning, wind gusts). However, the impacts strongly depended on the type of event and the type of sports as well as the time of the event and its location. For example, events that usually take place in the summer months between June and August were often affected by extreme heat, while events in spring or winter were affected by heavy rain or storms.

The organizers of one of the UEFA EURO 2024 fan zones particularly planned for extreme heat and heat stress-related incidents. The interviewee explained:

Our city center here … there’s heat accumulation, there’s little wind. And that’s all suboptimal, of course. If it’s 40 degrees again like last summer, we will have problems here and have therefore already thought about a few things. And of course, we just hope that it won’t be 40 degrees in mid-June because fewer visitors will surely come to the fanzones or they won't stay on site for so long if it is that hot.

On the other hand, the Bundesliga Football Club Werder Bremen is strongly affected by flooding due to its location next to the river Weser (see also, 2). As such, the interviewee from Werder Bremen highlighted:

… there is actually a very close involvement with flooding and flood protection concepts here, … which we regularly deal with and optimize. And now we were lucky again a few weeks ago, so to speak, when the water of the Weser was rising again, leading to partial flooding in our neighborhood. These are always aspects that we can learn from.

Depending on their locations, other Bundesliga clubs may not face flooding issues but could encounter different challenges. In the context of heat, one participant stated:

So the Bundesliga has a break for the summer. Ultimately, there’s also the question of how the weather and climate will develop. So maybe the Bundesliga will have to start later because August is still far too hot? I don’t know. At the moment, I think we’re probably still doing reasonably well as far as heat is concerned.

The international horse-riding event in May, in contrast, particularly deals with torrential rain incidents which particularly affect the parking areas:

Our car parks are all on pastureland. This year we were a bit flooded and then of course at some point, it becomes very difficult …. And we have to work with tractors, and usually pulling everyone in and then after the event pulling everyone out again…, so that’s a huge logistical challenge and also the additional costs, so hiring additional tractors, paying the drivers, etc. … and this happened the last two or three years.

Torrential rain generally leads to a variety of issues, as outlined by the employee of the event technology company:

Smaller events in particular are often hosted on grassy areas that are then simply no longer accessible at all after long periods of rain or can only be accessed leaving major damage. We’ve had that last year at almost every event and a few times this year too, which ultimately leads to higher costs. And I’ll be honest, unfortunately, we had to pay for these costs ourselves …. We actually have now started to include this in our terms and conditions for stage constructions, so for example, delays due to weather are actually at the expense of the client. Because we’re not talking about half an hour, concerning delays, but more like 5, 6 h and if you’re working with 10 people, you’re quickly a few thousand Euros short. That is one problem, and the second problem is who actually pays for the damage to public land in the long-term?

The second problem particularly pertains to events that take place on public land or in public areas and leave long-term damages, as for example, to lawns in public parks or public pools when trucks or other vehicles leave marks and damages due to the area being muddy and unstable.

Snow was also mentioned as an impact by several interviewees. In the context of football (soccer), the winter of 2023/24 with large amounts of snow heavily disrupted amateur leagues in Germany. For example, the Schleswig-Holstein Football Association (SHFV), an association in the northern part of Germany, had to cancel all games in all amateur leagues for two weekends in a row in November and December 2023. All age groups were affected. The reasons for these cancellations did not only include poor pitch conditions but also safety concerns for the spectators. These weather-related match cancellations disrupted the entire match and league schedule and negatively affected planning security for teams. Most teams and players, however, did not criticize the long break caused by the cancellations of several matches, but rather the tight program that followed—due to the many follow-up games needed. In stark contrast, the Special Olympics National Winter Games (900 athletes, 10 types of sports) in Thuringia in January/February 2024 were affected by a lack of snow. The interviewee stated:

… the time window in which you can organize the event and the regions in which you can organize it to have a high chance of guaranteed snow is getting narrower and narrower.

Storm, wind gusts as well as lightning and thunderstorms also present increasing impacts. This does not only affect the event itself but also the travel to and from the event, as one participant explained:

We have a football club here where we organize a huge tournament, which got cancelled because it was too dangerous for us to send the children on the trip in a heavy storm. So things like that happen too. So how do I even get to the actual event? Whether it takes place indoors is then actually of secondary importance. So at the moment when the weather situation is so extreme, that there is also the problem of travelling to the event, of accessibility.

Several interviewees reported incidents of tents collapsing in fierce winds, which points to the need to use robust and weather-resistant materials. In an events context, so-called “temporary structures” such as stages, canopies, and grandstands, play a significant role and need to be erected and operated in accordance with specific standards, regulations and laws. In Germany, temporary structures must comply with the general requirements of building law following section 3 of the Model Building Code (MBO): “Structures must be arranged, erected, modified and maintained in such a way that public safety and order, in particular life, health and the natural foundations of life, are not endangered” (190). There are four regionally defined wind zones and also four terrain categories, such as “open land without obstacles” or “urban areas”. These local conditions of the intended installation area must be considered when planning. For example, at wind speeds of 15 meters per second, i.e., wind forces of 7–8, all event operations must be stopped, and the side cladding must be removed so that the structure can be left standing safely even in case of a storm. However, organizers of smaller events might face problems in this context since they often do not have the knowledge about materials and equipment on the one hand, or of rules, regulations, and laws on the other. This could be, for example, “the sports club that sets up its DIY pavilion and sells cakes underneath” or the event sponsor who arrives “with a wobbly drop tent or pavilion with the company logo on it and is surprised that it flies away”. One interviewee therefore stated:

… something has to happen in that area. But that can only be done by the event organizer, right? The building authorities don’t have this on their radar, there is no legal regulation for it because the structures are too small.

In addition, sports infrastructure, especially outdoor facilities, are often not sufficiently protected against the impacts of climate change. Damage caused by flooding, or storm damage can significantly restrict the use of sports facilities and lead to long downtimes. What exacerbates this problem is a renovation backlog. Many sports facilities in Germany date back to the 1960s–1980s. These facilities need to better adapt to climate change, for example through shading, greening of façades, or rainwater storage and derivation.

A final impact highlighted was the increase in infections and diseases. As such, several smaller event organizers reported issues due to the growing populations of oak processionary moths, with one organizer even having to cancel their event because of health risks and council directives. The canoe association reported worsening water quality and an increase in neophytes, such as giant hogweed, as well as blue-green algae, resulting in frequent health problems for the athletes (e.g., diarrhoea).

### 4.3 Measures implemented to adapt to climate change

Protecting athletes, spectators and other participants against extreme heat, high temperatures and strong solar radiation played a very strong role for the interviewees. Several organizers have already postponed start times to earlier or later times of the day or are at least considering this as part of contingency plans to offer athletes and spectators better conditions. Shading—both natural and artificial—is also at the center of attention, particularly for grandstands and spectator areas. For the Special Olympics, the organizers planned to install 18 water fountains. However, only two could eventually be installed due to issues with water hygiene and required regulations. Still, with the help of fire services and the technical relief organization (THW) a reliable water supply system was made available during the event to guarantee the safety and well-being of all involved.

The Sports Medicine Commission from the German Football Association (DFB) has also published some specific recommendations for amateur football (soccer) matches (see Table 1), which include the postponement of the match (to the evening hours or another date) at more than 35 degrees Celsius (or more than 32 degrees Celsius with humidity above 80 per cent), drinking breaks, cooling aids (such as ice packs or cold towels), and additional water supply for players and spectators (DFB, 2024).

The two major events, Special Olympics and UEFA EURO 2024 particularly planned for a wide variety of measures to counteract the negative impacts of extreme heat. The interviewee from the UEFA EURO 2024 fan zone explained:

… from shading to water dispensers. How do we provide for people in an emergency if they can no longer get their drinks at the catering stands? That’s also a worst-case scenario if it’s super-hot and they have to queue for ages. That means we need lots of drinking water dispensers and information about the drinking water fountains, with maps of where to find them.

The adaptation measures also included discussions with caterers, who are often concerned that providing free drinking water might adversely affect their sales. As such, water dispensers were installed throughout the city but not within the official public screening areas. In addition, a large variety of “free refill areas and stations” (https://refill-deutschland.de/) were made available, and shops, cafés and restaurants participated in providing free water. These areas were promoted via the website and the event app. To ensure the availability of shaded areas, parasols and awnings were used, but also additional lounging and seating areas or “green islands” were hired and installed. The latter are made of sustainable materials and combine seating or lounging with integrated plants and water storage systems, thus intending to create a “green oasis” in the city while at the same time reducing the heat. The event website also listed a variety of buildings and structures within the city center that provided shelter from the sun, such as churches or bridges. Furthermore, the organizers intended to encourage visitors to put on sunscreen and hats and promoted the latter as giveaways to event sponsors. The interviewee explained:

We want to distribute as few items as possible that will end up in the rubbish afterwards. But some of the sponsors will hand out something anyway and then we encourage them not to hand out plastic stuff, but useful things like caps, cooling aids and the like.

The organizers also partnered with the German Federal Office for Radiation Protection to equip the host city with sunscreen dispensers around the city.

In the context of heat, it is not only important to protect athletes and spectators, but also volunteers and other event staff members. Communicating the risks of extreme heat and UV radiation to these groups and distributing protective materials (e.g., hats or caps, parasols, sunscreen) is therefore equally important. The interviewees from the event technology company explained:

That’s an occupational health and safety perspective that you now have to take, … for example, we’re now equipped to provide our employees with protective clothing against the sun and heat.

In the context of flooding, the Bundesliga Club Werder Bremen has set up cross-departmental working groups to deal with the impacts of flooding and other extreme weather incidents. They also closely cooperate with various partners, including the city council and other authorities (such as dike associations, the stadium owner, etc.) given that the issue is complex and does not only concern the stadium itself but also the surrounding areas and dikes. Extensive flood protection measures were completed in 2016 by the stadium owner, which included a 6.50 m U-shaped sheet pile wall and a pump system to remove rising groundwater in the event of an emergency with total costs of 5.6 million Euros (191). The stadium has since faced several flooding incidents again—for example in December 2023 during hurricane “Zoltan” when the sheet pile walls acted as valuable protective measures around the arena. The club plans to raise more awareness of climate adaptation and the associated risks, in particular related to flooding. This will be done both internally and externally to inform and involve all stakeholders.

Other measures to counteract torrential rain and flooding incidents include the provision of shelters and nearby buildings. In football, the use of artificial turf pitches is also seen as a way to minimize the impact of heavy rain and other extreme weather conditions. These pitches make it possible to play sports even in unfavorable weather conditions. From a construction and event set-up point of view, steel plates and wood chips are used to ensure the accessibility of event venues and grounds without leaving major damage or getting stuck while at the same time ensuring stability to temporary structures.

Since sports event sites often contain all kinds of tents and temporary structures, they are particularly vulnerable to storms and wind gusts. As such, the interviewees point to the importance of being prepared—which includes preparedness by volunteers and all event staff. One interviewee noted:

We have to ensure that things like advertising banners are actually taken down when there are heavy storms. Because … otherwise the construction fences will be knocked down. So we plan for the extreme case of having to evacuate places in certain situations…. And then how do you get these masses of people quickly to leave the city center in about half an hour? You have to accommodate them somewhere.

As such, having shelters available as well as a clear evacuation and emergency plan with well-defined responsibilities is crucial.

In the context of snow, several regional football associations in Germany have already reacted to the increased impacts and have adapted their match schedules and seasons. For example, the season of the Northeastern Football Association is set to start earlier, with the ball rolling again from the end of July. There will also be more matches in the first part of the season and the winter break has been extended by a few days, with play set to resume at the end of January (192). In doing so, it is hoped that there will be fewer cancellations or that cancellations will not have major impacts due to “buffer days” being available. However, it remains to be seen whether these measures will be effective or potentially counterproductive, as they may introduce new challenges such as heat-related issues with a new season starting in July already.

The scarcity of snow is also addressed. For the Special Olympics National Winter Games in Thuringia, for example, the organizers added new types of sports due to low levels of snow:

So we compensated a little by promoting the Winter Games as a multi-sports event, a major multi-sports event, with more and more weather-independent elements. So, for example, this time the program included dancing. This time we had indoor climbing on offer to diversify the sports program a little.

Including “buffer days” in international competitions is a measure introduced by several organizers since it allows flexibility to reschedule events when conditions are unsafe. This is particularly important for sports that are heavily dependent on weather conditions, such as sailing or open water swimming. In the context of the Special Olympics in Berlin in 2023, the interviewee highlighted:

We had an individualized approach according to each type of sports. And the types of sports that had a higher risk deliberately had buffer days built into the schedule that could or could not be used.

It is therefore vital to consider the unique environment of each sports type and venue, as each has different challenges and requires tailored responses to climate change.

Another very important measure to adapt to the impacts of climate change is weather monitoring with the help of different weather apps. The organizer of the Special Olympics also remarked:

We have worked with a meteorological company, based in Berlin, with whom we basically concluded an agreement and received a weather forecast from them twice a day, which was also location specific. Plus a 24 h hotline availability.

However, smaller local and regional events might not have the budget for this kind of collaboration and service and are dependent on regular, free weather apps. However, most interviewees agree that weather monitoring can also be done with a low budget. What is most important is to establish clear responsibilities and continuously monitor the weather, reacting swiftly and with adequate notice. In the event of an imminent threat, such as thunderstorm cells, the event team should convene regularly (e.g., every 15 min) to evaluate the situation and determine the appropriate response. Preparation is crucial and should involve not only the event team but also athletes, coaches, sponsors, volunteers, spectators, and other stakeholders. Everyone should be prepared and know what to do. This requires constant and clear communication and the development and implementation of emergency scenarios and risk management strategies. Constant review and adjustment of existing plans as well as clear guidelines on when competitions should be cancelled or postponed are also needed.

Finally, the important role of sports associations and federations is also highlighted frequently. They can offer support in emergencies, for example, by providing alternative sports grounds or helping to find funding when natural disasters affect events. The associations can also provide recommendations and guidelines to prepare for extreme weather conditions. In this context, the German Olympic Sports Federation (DOSB) has lately been instrumental. At the end of 2023, for example, they published a special brochure on climate protection and adaptation which was sent out to members as part of a newsletter. They also initiated the “Bodenheim Symposium”, a specialist conference at the end of 2023, during which sports associations, clubs and experts came together to discuss climate adaptation in sports and possible adaptation measures. The DOSB website also provides information on a large number of climate-related health risks (e.g., heat, extreme weather, UV radiation, etc.) and possible adaptation measures in sports, together with helpful links and information from scientific institutions. The German canoe association has launched the initiative “Kanu-morgen” (“canoe tomorrow”) with a dedicated website and brochure providing specific climate protection and adaptation measures related to sports facilities, venues and grounds, water bodies, individual paddlers and events (193).

### 4.4 Challenges involved for event organizers

Asked about the challenges involved, participants pointed to the lack of awareness and understanding of the differences between climate protection and climate adaptation, both within the general population but also in companies. It is also a challenge to integrate both climate protection measures and adaptation strategies into the existing structures and processes of sports organizations. Up to now, the focus has often been on climate protection, while adaptation to climate change has only recently received more attention. In addition, there are different opinions on climate change among people involved and not all stakeholders are aware of the urgency to act and implement measures. This can hinder the implementation of necessary measures and changes.

A particular challenge that all interviewees highlighted is the costs involved. In the future, sports event organizers must expect higher costs due to additional measures needed to be put in place or to repair damage. This includes, for example, extra human resources or additional equipment but also investment in infrastructure. One interviewee noted in this context:

… when sports facilities and stadiums are damaged by water, so like flooding, then of course it also becomes financially difficult. One of our shooting clubs here was affected. The recently renovated premises were again completely flooded, and they suffered a six-digit damage. So, no training, no competitions, no events anymore.

The costs to hire an event technology company will also increase given that weather-related delays in setting up and dismantling events will lead to an increased workload and technical requirements are becoming more complicated due to the changing weather conditions. These financial burdens can jeopardize the profitability of events. It was also emphasized that many actors in the industry are not adequately prepared for the future challenges of climate change. As such, the sports industry is still relatively inexperienced when it comes to climate adaptation and there is a need for more information, training, and resources to support associations and clubs in the implementation of adaptation measures. Due to the lack of resources and knowledge, many organizers approach the challenges in a rather casual way, often not being fully aware of the risks and consequences. One respondent noted:

Sometimes the communities themselves are the organizers, or the city marketing departments or a small sports club … and then it’s just done in a casual way. Where actually the regulatory offices or building authorities should intervene … and that is not done, there’s simply no consultation.

Determining who is responsible for damage and delays caused by extreme weather is becoming increasingly complex. This can lead to legal disputes and uncertainties.

In this context, sponsorship also involves several challenges and aspects to be considered. For instance, sponsored items brought to the event, such as tents and pavilions, may be branded with the sponsor's logo but fail to meet safety standards. Distributing products (e.g., branded sunscreen) can also be problematic since it may infringe on sponsorship rights. Similarly, offering free water during extreme heat conditions may upset caterers and sponsors who wish to sell their products. As such, integrating even smaller climate adaptation measures might lead to new issues and questions. One interviewee highlighted:

So just with these sunscreen dispensers… when it comes to cosmetic products, you always have to mention the producer, the distributor, and the ingredients. And some events are not allowed to mention brand names unless they are sponsors. So yes, that makes a lot of things more difficult. And then there are the costs. And you also have to worry about things like vandalism or how and when to refill them.

Overall, the findings highlight the need for event organizers to continuously adapt to changing climatic conditions, which requires additional resources. The challenge is to find suitable solutions that are easy to implement to hold future events safely and successfully. Overcoming these challenges requires proactive planning, adaptability and a clear understanding of the risks associated with extreme weather conditions.

### 4.5 Outlook and the future of climate adaptation in sports event contexts

The interviewees frequently pointed out that local authorities and smaller sports event organizers often do not have the necessary resources or infrastructure to respond appropriately to climatic changes. One interviewee remarked:

The smaller the community, the less expertise we have in the authorizing authorities on the subject of safety concepts, so there is nobody who can really adequately check the safety concept in this respect in case of doubt and then there’s therefore also nobody to point the finger at some issues and says to the organizer ‘Watch out, this doesn't work at all or it’s not possible under building law’ or something like that.

Thus, all interviewees agreed that more awareness needs to be built within the sports and events industries on climate adaptation and more training and further education needs to be offered. Asked what is needed in the future to better adapt sports events to climate change, one organizer referred to “constant education and awareness-raising.” Another one responded:

Greater sensitivity is required … a stronger focus on organizational measures. Better monitoring. Clear responsibilities, clear communication channels, which are not only relevant to the weather but are relevant to all safety issues.

Weather conditions will likely become even more unpredictable in the future which makes it difficult to make long-term plans. As such, organizers must be flexible and need to be able to react quickly to changing weather conditions and the challenges involved. Costs will continue to be one of the most important challenges in the future. One interviewee highlighted:

Insurance, technical expertise for organizational measures, technical expertise for structural measures, implementation of higher standards for structural measures. This all costs money. This will lead in the short, medium, and long-term, to outdoor events simply becoming more expensive. We have already noticed that. It’s not just inflation and the after-effects of the coronavirus, but I would say that we’re already noticing this in the area of temporary infrastructure because the requirements are increasing.

Finally, it is continuously emphasized by all participants that more measures need to be suggested, tested and applied and these need to be tailored to the specific type of sports, the size of the event and the specific environment within which the event operates. There is “no ‘one fits all’ approach.” The measures also need to be implementable without major investments. As such, several event organizers pointed to the different sports and event associations for further help.

It would be great to get some clear guidelines …. So, like specifically for our … events. Like some examples of some easy-to-implement measures that I could give to my staff or our volunteers, … that have already been tested by other event organizers. Or maybe some clear indicators. Like ‘if this happens then we recommend you do x.’ … Or perhaps just an exchange of ideas, like a ‘what have you done here, how did it work?’-kind of conversation.

In the future, sports event organizers must develop long-term strategies to adapt to the changing climatic conditions, thereby considering specific indicators and policies for the different impacts and the different circumstances. This—as one participant summarized—requires “resources, planning, collaboration, investment and possibly also changes to the infrastructure.”

## 5 Discussion and implications

This study has set out to investigate how sports events are affected by climate change (research question #1), what specific measures exist to adapt to the impacts (research question #2) as well as the overall status quo of climate adaptation in the German sports events industry (research question #3). With the help of an extensive literature review and 13 semi-structured expert interviews, the specific impacts of climate change, particularly through extreme weather incidents have been identified and analyzed. Similarly, a wide variety of measures—in response to each single impact—has been described and outlined. A detailed overview and summary of these measures, derived from both the theoretical (including a review of 227 research articles) and practical findings of this paper, is presented in the Supplementary Appendix Tables 4–8. These tables can help sports event organizers consider adequate adaptation measures according to what is reasonable and appropriate for their events and environments. The tables thus seek to provide hands-on options and guidance.

However, while a large variety of measures has been identified and included in these tables, these are by no means complete and can only provide some generic recommendations [in line with (129)]. As highlighted by the interviewees and also in the literature [e.g., (152)], these measures need to be tailored to the characteristics of the sports and the event and should also consider the specific environment and region in which these events take place. Sports event organizers (of specific types of sports) and sports associations should therefore collaborate to determine the needs and requirements of the respective sports type (including special equipment needed, special risks and other peculiarities pertaining to the sports and event type).

What has become evident is that sports event organizers have only just started to deal with the consequences of climate change—in line with the findings by Werner, Griese and Hoth (10). While measures exist and have been introduced, there is no strategic, long-term approach. In most cases, single measures are implemented in an ad-hoc manner to quickly deal with issues as they arise. These measures often emerge as a result of firsthand experience or an initial impact. For example, one organizer, after experiencing extreme temperatures on the day of their event, implemented a range of heat-reducing measures for the following year, only to be faced with torrential rain instead. Other organizers have simply not yet been affected by climate change and do therefore not see the need to introduce any measures. They rather take a precautionary, reactive approach, thus mirroring the findings by Kay and Vamplew (158).

Apart from the two major event organizers (Special Olympics, UEFA EURO 2024) who planned for a wider variety of measures, the other organizers often only referred to a mere handful of measures. Weather forecasting and monitoring with the help of simple weather apps (available on any mobile phone) appears to be the only measure used by all. Also, no specific indicators or threshold values (such as certain temperature, water, or air quality indices) have been used as a guideline or basis for any decision-making—for example, to interrupt or cancel an event. The literature points to several quite specific indicators, in particular related to heat [e.g., (22, 48, 151)]. As such, several sports associations or single event organizers use thresholds such as the WBGT (see also Table 1). However, at the more grassroots levels, organizers seem to make their decisions on an individual case-by-case basis and according to their subjective perception, i.e., what they perceive to still be a safe environment for all involved.

In the context of the Olympics and other major sports events, Racinais et al. (152) criticized the large variety of single policies developed by different federations, which use different indicators or are scaled in confusing formats and are therefore difficult to understand or implement in practice. This situation makes it particularly hard for event organizers and—even more so—for volunteers and other event staff. Most of the proposed guidelines are aimed at elite athletes and professional sports events, which benefit from access to professional medical support and services, as well as substantial financial resources. What also needs to be considered is that temperature thresholds for professional athletes are different compared to those for the general population (e.g., spectators, volunteers), yet, the organizers need to cater for all (150). It is therefore crucial to develop climate adaptation policies for sports events that are efficient, understandable, realistic, and implementable. These policies should also be applicable at the grassroots level, enabling smaller sports event organizers, event staff, and volunteers to easily and quickly implement them. For instance, the Union Cycliste Internationale (194) provides a “practical guide for estimating the WBGT index”, which details the application of the WBGT index step-by-step, including screenshots and a calculation table. To support such initiatives, educational institutions, universities, sports and event associations, and federations could establish appropriate training programs, seminars, and webinars.

In addition, the policies so far have focused very much on heat-related impacts, while other impacts have not received the same attention [e.g., (6, 22, 33)]. As such, developing a policy with clear recommendations for *each* relevant impact (heat, air quality, UV radiation, etc.) is needed. This policy should be validated by experts in each field such as meteorologists, medical doctors and sports scientists, experienced event managers, event technicians, insurance companies, etc. and should—once again—cater to the special characteristics of the sports, the event and the athletes (e.g., age and acclimation status) (152). Ideally, funding should be made available by governments or sports and event associations to work on these specific policies.

The findings have also demonstrated that increasing costs are a major issue. These costs include investments into the infrastructure, special equipment, additional human resources as well as training and further education initiatives. In this context, upgrades or refurbishments (after damage) of buildings and sports facilities are particularly costly and may strongly affect small sports event organizers. In addition, insurance premiums may rise. All these costs will affect and burden sports event organizers in the future. This could raise concerns that smaller organizers may no longer be able to host events due to insufficient financial and human resources, highlighting the need for increased support from governments, sports and event associations. In this context, Kay and Vamplew [(158), p. 103] warned that “… it is probable that only the wealthiest organisations could afford to weatherproof facilities while the majority of clubs, councils and schools would still have to face the elements.” In this context, it should be emphasized again that the operational side of adaptation measures depends on the capacity of sports event organizations to successfully implement them (55). Therefore, it is important to demarcate the notion of adaptive capacity that is needed for events and assess the extent to which organizers are able to adapt as well as to identify the knowledge gaps that constrain the adaptive capacity of events.

Given the increasing unpredictability of the weather conditions in the future due to climate change (195), event organizers need to become even more flexible in the future to be able to react and address sudden changes. They need to be well prepared for the unknown. As knowledge and information continue to grow from mega and major events, as well as elite athlete contexts, there is significant potential to share this expertise with smaller sports and event organizations, enabling them to learn and implement best practices. Additionally, event organizers should secure appropriate insurance to cover costs resulting from climate change-related impacts or in case the event needs to be interrupted, postponed, or cancelled. Closer cooperation with insurance companies is therefore also recommended.

As part of a critical discussion, it also needs to be mentioned that sports events are not just a victim of climate change, but also an important contributor—in particular the large-scale events (2, 110). Goldblatt [(2), p. 3) highlighted: “If the sports world is to make its own contribution to climate change action, then it needs to acknowledge its own role in creating the problem and radically reduce its carbon footprint.” However, previous research has demonstrated that most sports and sports events’ environmental commitment and governance are still at a low level (2, 196, 197) and more needs to be done to reduce the sports events industry's carbon emissions. Fewer sports events and competitions held less often, might thus be part of the solution. In addition, climate protection and climate adaptation should be addressed concurrently to maximize their synergies.

Finally, the review of the literature has demonstrated that there is a vast range of scientific studies on the various impacts of climate change on different types of sports [e.g., (50, 198, 199)], particularly from the fields of sports medicine, health, and climate science. However, the sheer volume of information can be overwhelming, making it difficult to grasp the most critical aspects. Therefore, there is a need to better organize and collate this extensive knowledge, making it more accessible and implementable to those working on the front lines.

## 6 Limitations and future research

This study used a qualitative approach to explore social real-life phenomena in sports events contexts. The qualitative approach has been criticized for its subjectivity and lack of replicability, generalizability, and transparency (200). Although the qualitative findings of this study may not be widely generalized, they can be used to inform future research (182). In addition, it must be acknowledged that the sample is relatively small. However, this assisted in exploring the experiences and perceptions of the interviewees in more detail and depth. The sample also only included the organizers from certain types of outdoor sports such as football, cycling, horse riding or triathlons as well as from selected multi-sports events. The findings can thus not be regarded as representative of the entire German sports event industry. Further studies should also include indoor event organizers and how they are impacted by climate change (e.g., through travel disruptions or disruptions of supply chains). As such, future studies with more diverse and larger samples are needed to validate the findings. Finally, further research on the adaptation measures would be of value. For example, it would be helpful to identify further adaptation measures in other sports events-related contexts, thus continuously expanding the tables in the Appendix. In addition, further conceptualization and categorization of the measures is needed. The current categorization is based on Schneider et al. (129) and only focuses on structural/physical, organizational, communication and legal/collaboration-related measures. Future research could add further consideration to and analysis of different dimensions (e.g., structural, physical, social, institutional, etc.) and situate them in a broader context. Despite these limitations, the study can provide valuable insights into climate adaptation challenges of German sports event organizers, thus enhancing our understanding of important contexts and interrelationships and therefore assisting to better address these challenges in the future.

## Data Availability

The data is available upon reasonable request from the corresponding author.

## Footnotes

^1^The Representative Concentration Pathways (RCPs) describe four different 21st century pathways of greenhouse gas (GHG) emissions and atmospheric concentrations, air pollutant emissions and land use. RCP8.5 is the scenario with the highest GHG emissions; see Intergovernmental Panel on Climate Change, 2014.
