# Supplementary material for: The need to (climate) adapt: perceptions of German sports event planners on the imperative to address climate change
Source: Front Sports Act Living. 2024 Dec 23;6:1505372. doi: 10.3389/fspor.2024.1505372 (PMC11700741; doi:10.3389/fspor.2024.1505372)
Supplement: Supplementary file 1 [file Table4.pdf]

air quality - potential responses and measures related to:			
**event location / venue / facilities**	**organisational processes**	**communication processes**	**legal / regulations and collaboration**
assessing the location's environment and related risks associated with air quality (e.g. urban vs. rural areas)	continosly measuring the air quality including pollen concentration (with one team member designated as responsible)	information material/display boards/announcements to inform spectators/visitors, athletes and all other stakeholders about the actual air quality and potential health consequences	determining the exact metrics/threshold values (e.g. using the air quality index, AQI) that will be used to apply certain measures (i.e., when to do what)
choosing locations with a low proportion of trees and plants that enable allergies (e.g. birch, hazel, alder)	limiting outdoor exercises during high pollution periods (e.g., through longer breaks, interruptions)	information material/display boards/announcements to inform spectators, athletes and all other stakeholders about contingency plans in case the air quality reaches a certain threshold	determining changes in scoring or performance metrics to account for the challenging conditions (in collaboration with the specific sports association/federation)
	distributing N95 masks; installing oxygen cylinders in dressing areas	immediate communication of an alternative date/time and clear policies as to what will happen to scoring in case the event is interrupted or cancelled (to minimize pressure/mental load on athletes and ensure transparency)	close collaboration with emergency services (including police, fire services, disaster response teams, etc.)
	distributing antioxidant supplements		collaboration with meteorologists, weather and climate experts, medical doctors and medical institutions (including dermatologists, psychologists and other sports medicine specialists), councils, transport and regulatory authorities, etc.
	continuous weather forecasting and monitoring (with one team member designated as responsible), including the assessment of threshold exceedances		partnerships between and among clubs, sports venues, sports federations and associations or sports event organizers for mutual support
	postponing the event to a different day or season		continuous training and further education of event organizers, staff, volunteers, council staff and all other stakeholders concerning the impacts of bad air quality and potential adaptation measures (tailored to the event)
	changing the location of the event		
	moving indoors (if possible)		
	cancelling the event		

extreme weather - potential responses and measures related to:			
**event location / venue / facilities**	**organisational processes**	**communication processes**	**legal / regulations and collaboration**
avoiding buildings/venues that are located in areas that are at risk of flooding; if applicable: using flow water and heavy rainfall maps (available from local councils) to identify potential risk areas	continuous weather forecasting and monitoring ((with one team member designated as responsible); cooperation with professional weather advice services	information material/display boards/announcements to inform spectators/visitors, athletes and all other stakeholders about the actual conditions	in line with applicable local laws/regulations: having a detailed emergency/evacuation plan ready (including clear responsibilities and communication strategies)
continuously assessing the event venue and its surroundings to identify risks (including hazards from fallen trees, holes, uneven surfaces, etc.)	installing additional shelters to protect all event participants	information material/display boards/announcements to inform spectators/visitors, athletes and all other stakeholders about contingency plans	determining the exact metrics/thresholds that will be used to introduce certain measures (i.e., when to do what)
availability of shelters in close proximity (public buildings like schools, gyms, or additional tents, etc.) in case of thunderstorms, torrential rain.	using weather-proof material and equipment at all times and in all situations	immediate communication of an alternative date/time and clear policies as to what will happen to scoring in case the event is interrupted or cancelled (to minimize pressure/mental load on athletes and ensure transparency)	determining changes in scoring or performance metrics to account for the challenging conditions (in collaboration with the specific sports association/federation)
choosing buildings with rainwater storage, harvest or drainage systems	interrupting the event		close collaboration with emergency services (including police, fire services, disaster response teams, etc.)
	postponing the event to a different day or season		collaboration with with meteorologists, weather and climate experts, medical doctors and medical institutions, councils, transport and regulatory authorities, etc.
	moving indoors (if possible)		partnerships between and among clubs, sports venues, sports federations and associations or sports event organizers for mutual support
	cancelling the event		continuous training and further education of event organizers, staff, volunteers, council staff and all other stakeholders concerning the impacts of extreme weather and potential adaptation measures (tailored to the event)

(forest) fires - potential responses and measures related to:<br>event location / venue / facilities	organisational processes	communication processes	legal / regulations and collaboration
assessing the location / venue and its risk potential to be affected by forest fires	continuous weather forecasting and monitoring with a particular focus on air quality monitoring (one team member designated as responsible); cooperation with professional weather advice services	information material/display boards/announcements to inform spectators, athletes and all other stakeholders about the actual situation and conditions	in line with applicable local laws/regulations: having a detailed emergency/evacuation plan ready (including clear responsibilities and communication strategies)
continuously monitoring/checking the event venue and its surroundings to identify risks associated with forest fires	with deteriorating air quality: see measures for air quality	information material/display boards/announcements to inform spectators, athletes and all other stakeholders about contingency and evacuation plans	close collaboration with emergency services (including police, fire services, disaster response teams, etc.)
	changing the location of the event	immediate communication of an alternative date/time and clear policies as to what will happen to scoring in case the event is interrupted or cancelled (to minimize pressure/mental load on athletes and ensure transparency)	determining the exact metrics/threshold values (e.g. using the air quality index, AQI) that will be used to apply certain measures (i.e., when to do what)
	postponing the event to a different day or season		determining changes in scoring or performance metrics to account for the challenging conditions (in collaboration with the specific sports association/federation)
	cancelling the event		collaboration with meteorologists, weather and climate experts, medical doctors and medical institutions, councils, authorities, transport and regulatory offices, etc.
			Partnerships between and among clubs, sports venues, sports federations and associations or sports event organizers for mutual support
			continuous training and further education of event organizers, staff, volunteers, council staff and all other stakeholders concerning the impacts of extreme weather and potential adaptation measures (tailored to the event)
