# Supplementary material for: The need to (climate) adapt: perceptions of German sports event planners on the imperative to address climate change
Source: Front Sports Act Living. 2024 Dec 23;6:1505372. doi: 10.3389/fspor.2024.1505372 (PMC11700741; doi:10.3389/fspor.2024.1505372)
Supplement: Supplementary file 2 [file Table5.pdf]

Extreme heat - potential responses and measures related to:			
**event location / venue / facilities**	**organisational processes**	**communication processes**	**legal / regulations and collaboration**
choosing microclimate-favourable locations/venues	offering (free) additional water, movable water dispensers, tanks or additional hydration stations	raising awareness of adequate clothing for athletes and spectators/visitors (e.g. light, breathable fabrics, long sleeves, sunhats, sunglasses, etc.)	introducing a "heat orientation plan" (see Schneider, 2024)
choosing venues/buildings with modern insulation and energy-efficient refurbishment (e.g. reflective colors)	postponing the start times (to the early mornings / evenings); establishing an extended break around noon ("siesta")	information material/display boards/announcements to inform spectators/visitors, athletes and all other stakeholders about the actual temperature and humidity and the potential health consequences	determining the exact metrics/thresholds (e.g., UTCI, mPET, WGBT, etc.) that will be used to introduce certain measures (i.e., when to do what)
choosing venues/buildings whose temperature can be managed (e.g., ventilation, air conditioning)	offering additional drinking / cooling breaks; extending the competition time to offer more drinking / cooling breaks (e.g., football: from 90 to 110 minutes regular play time); ; easing competition rules for extra water supply	information material/display boards/announcements to inform spectators, athletes and all other stakeholders about contingency plans in case temperatures reach a certain threshold	determining changes in scoring or performance metrics to account for the challenging conditions (in collaboration with the specific sports association/federation)
choosing venues/locations that offer both natural shading (such as trees and other plants) and artificial shading (including permanently installed options as well as movable parasols or awnings)	decreasing competition duration or race distances to reduce the athletic load and/or protect athletes and spectators; if possible, changing players/referees more often; potentially even changing the course (e.g., in loops around hydrating stations)	immediate communication of an alternative date/time and clear policies as to what will happen to scoring in case the event is interrupted or cancelled (to minimize pressure/mental load on athletes and ensure transparency)	close collaboration with emergency services (including police, fire services, disaster response teams, etc.)
choosing venues with green roofs/façades or venues/temporary structures (e.g. tents) in light colors such as white (the albedo, a measure of reflectivity, can help with the assessment. Solar energy is reflected back into space	providing free sunhats, umbrellas/parasols/awnings, fans and sunscreen to spectators and staff; providing adequate clothing to staff and volunteers		collaboration with public pools to offer refreshments to spectators, volunteers, and staff
choosing venues/locations that offer large cool areas, access to fridges/refrigerators/cold storage rooms	offering catering with waterrich fruits and vegetables (e.g. melons, cucumber) as well as salty snacks and carbonhydrating and mineral-containing drinks; reducing or not offering alcoholic beverages		collaboration with meteorologists, weather and climate experts, medical doctors and medical institutions (including dermatologists, psychologists and other sports medicine specialists), councils, authorities, transport and regulatory offices, etc.
choosing locations/venues that offer water dispensers, water tanks or accessible fire hydrants to be used for increased water supply (drinking quality needs to be ensured!); access to (temporary) shower facilities	providing additional cooling areas / tents; movable air-conditioners; access to fridges / cold storage rooms; additional retreat areas, tents or rooms		partnerships between and among clubs, sports venues, sports federations and associations or sports event organizers for mutual support

Extreme heat - potential responses and measures related to (cont'd): event location / venue / facilities	organisational processes	communication processes	legal / regulations and collaboration
choosing locations/venues that harvest, store and recycle water to save water	offering cooling aids pre/during/post event, e.g. ice packs/ice vests/ cold towels/hats/headbands/socks; (inflatable) ice tubes/baths; misting areas for spectators, athletes and staff; integrating existing water fountains in urban areas into the event		continuous training and further education of event organizers, staff, volunteers, council staff and all other stakeholders concerning the impacts of extreme temperatures and potential adaptation measures (tailored to the event)
availability of and access to stable buildings / shelters in close proximity in case of enduring heat/extreme temperatures	preparing "emergency give-away bags" (see Schneider, 2024) for all spectators/participants with a water bottle, sunscreen, lip balm, hat, sunglasses, insect repellent, disinfectants/sanitisers		
availability of defillibrators, wheelchairs, walkers,	medical services and medical monitoring for athletes; medical services and first aid stations for spectators and staff		
integrating air corridors into temporary structures (e.g. tents) to ensure the unobstructed flow of fresh, cool air.	logistical and re-programming considerations, e.g., to avoid queues or seating areas in the plain sun		
	hiring more staff / volunteers to increase break times and reduce working times in the plain sun		
	monitoring body temperatures (athletes, staff); (if possible) video surveillance to survey the event grounds and identify individuals in distress		
	continuous weather forecasting and monitoring, including the assessment of threshold exceedances (with one team member		
	ensuring the availability of (portable) defillibrators and wheelchairs		
	changing the location of the event		
	interrupting the event		
	postponing the event to a different day or season		
	moving indoors		
	cancelling the event		
