# Supplementary material for: The need to (climate) adapt: perceptions of German sports event planners on the imperative to address climate change
Source: Front Sports Act Living. 2024 Dec 23;6:1505372. doi: 10.3389/fspor.2024.1505372 (PMC11700741; doi:10.3389/fspor.2024.1505372)
Supplement: Supplementary file 3 [file Table6.pdf]

**infections and diseases - potential responses and measures related to:**			
**event location / venue / facilities**	**organisational processes**	**communication processes**	**legal / regulations and collaboration**
assessing the location's environment in relation to standing water areas (water barrels, puddles, ponds) to avoid a high level of mosquito population	offering food that is not easily perishable; hygenic checklists and desinfection facilities (particularly pertaining to food)	raising awareness of adequate clothing for athletes and spectators (e.g., long sleeves)	close collaboration with emergency services (including police, fire services, disaster response teams, etc.)
continuously monitoring/checking the event location/area to identify potential hazards (e.g. wasp nests, mosquitos, pests like ticks, oak procession moths or plants that encourage allergic reactions such as alder, birch, hazel)	distributing insect repellent; hiring technical solutions against mosquitos	information material/display boards/announcements to inform spectators, athletes and all other stakeholders about the actual conditions/potential hazards	collaboration with with meteorologists, weather and climate experts, medical doctors and medical institutions, etc.
strategically considering the positioning of temporary structures (e.g., tents, stages) in relation to local flora & fauna (e.g. proximity to wasp, mosquito, oak procession moths populations)	offering first aid stations and medical services (with a particular focus on wound infections, pests, insect bites, allergic reactions, etc.)	information material/display boards/announcements to inform spectators, athletes and all other stakeholders about contingency plans	partnerships between and among clubs, sports venues, sports federations and associations or sports event organizers for mutual support
	preparing "emergency give-away bags" (Schneider, 2024) for spectators with a water bottle, sunscreen, lip balm, hat, sunglasses, insect repellent, desinfectants/sanitizers	immediate communication of an alternative date/time and clear policies as to what will happen to scoring in case the event is interrupted or cancelled (to minimize pressure/mental load on athletes and ensure transparency)	continuous training and further education of event organizers, staff, volunteers, council staff and all other stakeholders concerning potential infections and adaptation measures (tailored to the event)
	continuous weather forecasting and monitoring including pollen movements (with one team member designated as responsible)		
	moving indoors		
	postponing the event to a different day or season		

**mental health - potential responses and measures related to:**			
**event location / venue / facilities**	**organisational processes**	**communication processes**	**legal / regulations and collaboration**
availability of retreat areas / rooms / tents, etc.	preparing additional retreat areas /room / tents	information material/display boards/announcements to inform spectators, athletes and all other stakeholders about contingency plans	close collaboration with medical doctors and services, including psychologists
		immediate communication of an alternative date/time and clear policies as to what will happen to scoring in case the event is interrupted or cancelled (to minimize pressure/mental load on athletes and ensure transparency)	continuous training and further education of event organizers, staff, volunteers, council staff and all other stakeholders concerning impacts on mental health and potential adaptation measures (tailored to the event)

scarcity of snow/ice - potential responses and measures related to:			
**event location / venue / facilities**	**organisational processes**	**communication processes**	**legal / regulations and collaboration**
choosing a location that is located in higher altitude or in areas with favourable snow and ice conditions	changing the start times	information material/display boards/announcements to inform spectators, athletes and all other stakeholders about the actual conditions and potential hazards/risks	determining changes in scoring or performance metrics to account for the challenging conditions (in collaboration with the specific sports association)
continuously monitoring/checking the event area and its surroundings to identify risks/hazards (e.g. icy conditions, lack of snow/ice, increased levels of mountain and rock slides, rock fall, mud slides, avalanche risks, etc.)	reducing the competition/race time	information material/display boards/announcements to inform spectators, athletes and all other stakeholders about contingency plans	collaboration with meteorologists, weather and climate experts, medical doctors and medical institutions, councils, authorities (e.g. mountain watch and rescue services), transport and regulatory offices, etc.
	making use of technical solutions (e.g. artificial snow); snow farming; transporting snow from other areas to the event	immediate communication of an alternative date/time and clear policies as to what will happen to scoring in case the event is interrupted or cancelled (to minimize pressure/mental load on athletes and ensure transparency)	closer collaboration with emergency services (including police, fire services, disaster response teams, etc.)
	continuous weather forecasting and monitoring (with one team member designated as responsible)		partnerships between and among clubs, sports venues, sports federations and associations or sports event organizers for mutual support
	interruptiong the event		continuous training and further education of event organizers, staff, volunteers, council staff and all other stakeholders concerning the potential impacts and adaptation measures (tailored to the event)
	changing the location of the event (e.g. to higher altitude / areas with better climatic conditions); moving		
	postponing the event to a different day or season		
	cancelling the event entirely		
	substituting the event (e.g. with other tourism activities like hiking or other types of events)		
