# Supplementary material for: The need to (climate) adapt: perceptions of German sports event planners on the imperative to address climate change
Source: Front Sports Act Living. 2024 Dec 23;6:1505372. doi: 10.3389/fspor.2024.1505372 (PMC11700741; doi:10.3389/fspor.2024.1505372)
Supplement: Supplementary file 4 [file Table7.pdf]

UV radiation - potential responses and measures related to:			
**event location / venue / facilities**	**organisational processes**	**communication processes**	**legal / regulations and collaboration**
choosing locations/venues that provide natural (trees and other plants) and artificial shading (permanently installed but also in the form of movable parasols or awnings)	postponing the start times (to the early mornings / evenings)	raising awareness of adequate clothing for athletes and spectators, staff (e.g. light, breathable fabrics, long sleeves, sunhats, sunglasses, etc.)	determining changes in scoring or performance metrics to account for the challenging conditions (in collaboration with the specific sports association/federation)
	decreasing competition / race times to reduce the time spent under UV radiation for athletes, spectators and staff	information material/display boards/announcements to inform spectators, athletes and all other stakeholders about the UV radiation index and potential health consequences	determine the exact metrics /thresholds (e.g. UV index used by the German Federal Office for Radiation Protection) that will be used to introduce certain measures (i.e., when to do what)
	providing sunhats, umbrellas/parasols/awnings, fans and sunscreen	information material/display boards/announcements to inform spectators, athletes and all other stakeholders about contingency plans in case UV radiation reaches a certain threshold	close collaboration with emergency services (including police, fire services, disaster response teams, etc.)
	preparing "emergency give-away bags" (see Schneider, 2024) for all spectators with a water bottle, sunscreen, lip balm, hat, sunglasses, insect repellent, disinfectants/sanitizers	immediate communication of an alternative date/time and clear policies as to what will happen to scoring in case the event is interrupted or cancelled (to minimize pressure/mental load on athletes and ensure transparency)	collaboration with meteorologists, weather and climate experts, medical doctors and medical institutions (including dermatologists, psychologists and other sports medicine specialists), councils, authorities, transport and regulatory offices, etc.
	continuous weather forecasting and monitoring including the assessment of threshold exceedances (with one team member designated as responsible)		partnerships between and among clubs, sports venues, sports federations and associations or sports event organizers for mutual support
	postponing the event entirely to a different day or season		continuous training and further education of event organizers, staff, volunteers, council staff and all other stakeholders concerning the impacts of high UV radiation and potential adaptation measures (tailored to the event)
	interrupting the event		
	moving indoors		
	cancelling the event		

**water quality - potential responses and measures related to:**			
**event location / venue / facilities**	**organisational processes**	**communication processes**	**legal / regulations and collaboration**
assessing the event location's environment and related risks associated with water quality (e.g., inflows)	continously monitoring the water quality including the assessment of threshold exceedances (with one team member designated as responsible)	information material/display boards/announcements to inform spectators, athletes and all other stakeholders about the water quality and potential health consequences	establishing clear policies as to when to close waterways (including the use of specific indicators / thresholds)
consulting historic data of the water quality and previous issues	continuous weather forecasting and monitoring including the assessment of threshold exceedances (with one team member designated as responsible)	information material/ display boards/announcements to inform spectators, athletes and all other stakeholders about contingency plans in case the water quality deteriorates/reaches a certain threshold	determine changes in scoring or performance metrics to account for the challenging conditions (in collaboration with the specific association/federation)
	interrupting the event	immediate communication of an alternative date/time and clear policies as to what will happen to scoring in case the event is postponed or cancelled (to minimize pressure/mental load on athletes and ensure transparency)	close collaboration with emergency services (including police, fire services, disaster response teams, etc.)
	changing the location of the event		collaboration with with meteorologists, weather and climate experts, medical doctors and medical institutions (including dermatologists, psychologists and other sports medicine specialists), councils, authorities, transport and regulatory offices, etc.
	moving indoors (if applicable)		partnerships between and among clubs, sports venues, sports federations and associations or sports event organizers for mutual support
	postponing the event to a different day or season		continuous training and further education of event organizers, staff, volunteers, council staff and all other stakeholders concerning the impacts of bad water quality and potential adaptation measures (tailored to the event)
	cancelling the event		
