# Supplementary material for: The need to (climate) adapt: perceptions of German sports event planners on the imperative to address climate change
Source: Front Sports Act Living. 2024 Dec 23;6:1505372. doi: 10.3389/fspor.2024.1505372 (PMC11700741; doi:10.3389/fspor.2024.1505372)
Supplement: Supplementary file 5 [file Table8.pdf]

	Measures
**General/overarching measures**	investment into the infrastructure to make venues future-proof (e.g. more artificial turf pitches in football; more water dispensers/tanks, shaded areas, rainwater storage, rainwater derivation)
	investment in materials/equipment that are suitable and resilient in extreme weather conditions (e.g. tents, awnings)
	continuous site inspections and assessments to identify hazards
	new hosting models for mega-events (e.g. one host only for the Olympic Games or four to five host regions that rotate; see Orr, 2024)
	incorporating "buffer days" into international competitions to compensate for days when safe events cannot be held
	evaluating the supply chain of products and equipment required for the event to identify potential impacts of extreme weather incidents and develop alternative plans
	developing and establishing a specific long-term strategy with tailor-made measures for each event that considers the special characteristics of the sport, the characteristics of the event and its surroundings/environment as well as the characteristics of the athletes (e.g., age and acclimation status),spectators and other stakeholders (see Racinais et al., 2023).
**Addtional cross-sectional measures**	**Measures**
information, education & training	continuous training and education on the (potentially severe) impacts of climate change and the measures to adapt, addressing not only organizers, but also the general staff, volunteers, sponsors, councils, etc.
	continous awareness building: making participants, athletes and staff aware of the dangers, hazards and risks; increasing the awareness of personal responsibilities to keep safe (e.g. applying sunscreen, drinking water)
	providing constant information to all involved about the acutal conditions, the inherent risks and contingency plans in place (e.g. through flyers, display boards, websites,social media, apps, etc.)
	extensive knowledge management and transfer among all stakeholders involved; systematic documentation of all measures provided including their effectivness (lessons learned)
collaboration	collaboration with meteorologists and weather and climate experts
	collaboration with medical doctors and medical institutions (e.g. dermatologists, sports medicine, internal medicine, respiratory medicine, toxicologists, allergologists, infectiologists, psychologists, etc.)
	collaboration with councils, authorities and other relevant institutions (including public authorities, transport authorities, regulatory offices, urban and city planners, landscape gardeners, biologists, architects)
	collaboration with emergency services including police, fire services, technical aid services, disaster services
	collaboration with insurance companies
	collaboration with other clubs and venues for mutual support (e.g., offering alternative grounds/venues)
	collaboration with relevant associations (e.g. event associations, sports associations) for guidelines, support/advice, monetary support

Addtional cross-sectional measures	Measures (cont'd)
policies and plans	establishing and communicating clear policies as to when to interrupt, postpone or cancel the event including clear indicators/thresholds; clear and easy application and communication (e.g., using color-coded flag systems)
	having a detailed evacuation plan ready including clear responsibilities
	having a clear communication strategy ready that includes all stakeholders and all relevant contact details
	having clear policies in place concerning what happens to the competition and the scoring in case the event is cancelled/interrupted
	having adequate insurance coverage
	determining an alternative date in case of interruptions/cancellation that can quickly be communicated
	including climate adaptation measures into the event app, on the website as well as in communciation with athletes, spectators and staff
